# Mitochondrial Regulation of Ferroptosis in Cancer Cells

**DOI:** 10.7150/ijbs.105446

**Published:** 2025-02-24

**Authors:** Zilin Ding, Zhiyu Li, Kai Sun, Yi Liu, Zhou Fang, Shengrong Sun, Chenyuan Li, Zhong Wang

**Affiliations:** Department of Breast & Thyroid Surgery, Renmin Hospital of Wuhan University, Wuhan, Hubei, People's Republic of China.

**Keywords:** ferroptosis, cancer, mitochondrial metabolism, mitochondrial quality control

## Abstract

Ferroptosis is an iron-dependent nonapoptotic regulated cell death modality characterized by lethal levels of lipid peroxide accumulation and disrupted antioxidant systems. An increasing number of studies have revealed correlations between ferroptosis and the pathophysiology and treatment of cancer. Given the intricate involvement of mitochondria in ferroptosis, as suggested by previous studies, here, we review advances in understanding the roles of mitochondrial quality control and mitochondrial metabolism (including the roles of the TCA cycle, reactive oxygen species, iron metabolism, and lipid metabolism) in cancer-related ferroptosis and outline the molecular mechanism and clinical translation of mitochondria-related ferroptosis in cancer treatment. with the aim of promoting the precise utilization and prevention of ferroptosis in cancer therapeutics.

## Introduction

Ferroptosis is a nonapoptotic cell death modality characterized by lethal lipid peroxidation resulting from an imbalance in cellular metabolism and redox homeostasis, and it has attracted considerable attention in the scientific community because cells undergoing ferroptosis are morphologically different and the underlying mechanisms of ferroptosis differ from those of other known forms of regulated cell death, such as apoptosis and necrosis^1^. Recent studies have linked ferroptosis to various pathological conditions and diseases, and its important roles in cancer development and treatment are increasingly recognized^2^, ^3^. Notably, numerous types of therapy-resistant cancer cells, especially those that show high levels of mesenchymal and dedifferentiation characteristics are more susceptible to ferroptosis^4^, ^5^, ^6^, ^7^. Classic therapeutic agents, such as lapatinib and sorafenib, have been shown to induce ferroptosis and arrest tumor growth by increasing cellular iron levels or inhibiting cystine uptake^8^, ^9^. Therefore, the exploration of effective ferroptosis inducers or inhibitors may lead to a breakthrough in the search for treatments for cancer^10^.

Mitochondria are cellular energy powerhouses involved in maintaining cell survival, defending against oxidative stress, and promoting cellular metabolic homeostasis. On one hand, mitochondria are sites of oxidative phosphorylation and large amounts of energy production, which is needed for metabolic activity. On the other hand, mitochondria are the major organelles responsible for reactive oxygen species (ROS) production and host fatty acid metabolism, iron metabolism, and many other important metabolic processes^11^. The external structure and cellular location of mitochondria are critical for their function and depend on a highly regulated quality control system, which involves, for example, continuous fission and fusion that govern the overall shape and timely removal of damaged mitochondria through mitophagy^11^.

Given the central role of mitochondria in the regulation of cell death and the close association between ferroptosis and cellular metabolism, researchers have hypothesized that mitochondria may be involved in the regulation of ferroptosis. One of the characteristics of ferroptosis that initially distinguishes it from other types of regulated cell death is the difference in mitochondrial morphology, as observed via electron microscopy^1^. However, early studies revealed that cells with lost mitochondrial DNA or depleted mitochondria remained sensitive to ferroptosis^1^, ^12^, while other studies concluded that mitochondrion-depleted HT-1080 cells appeared to be less sensitive to ferroptosis triggered by cysteine deprivation but showed no significant resistance to RSL3-induced ferroptosis^13^. In summary, increasing evidence suggests that the role of mitochondria in ferroptosis is context dependent.

This review discusses the known roles of mitochondria in regulating ferroptosis, especially amino acid metabolism, iron metabolism, and fatty acid metabolism, and presents the conflicting data and plausible explanations for the role of mitochondria in the regulation of ferroptosis in cancer.

## 1. An overview of the ferroptosis pathway

Iron overload is closely related to ferroptosis. The iron-mediated Fenton reaction and iron-dependent enzymes lipoxygenase (ALOX) and cytochrome P450 oxidoreductase (POR) play vital roles in lipid peroxidation. These factors partially explain the iron-dependent characteristic of ferroptosis. Iron homeostasis is achieved through the strict regulation of iron uptake, storage, utilization, and export. Transferrin is an iron-carrier protein in serum that transports extracellular iron into cells by interacting with transferrin receptor 1 (TFR1), thereby forming the labile redox-active iron pool required for ferroptosis^14^. Intracellular iron levels can be sensed by intracellular metal sensors, which respond to initiate the regulation of the levels of iron storage and transport, change the labile iron pool level, and ultimately increase or decrease the sensitivity of ferroptosis^15^. Fe^3+^ is stored by ferritin, which is composed of the ferritin light chain (FTL) and ferritin heavy chain 1 (FTH1). Cytoplasmic ferritin levels can be downregulated by NCOA4-mediated ferritinophagy and the depletion of other autophagy-related genes, such as ATG5 and ATG7^16^, ^17^, ^18^. In addition, dihydroartemisinin has been shown to sensitize cancer cells to ferroptosis by inducing the lysosomal degradation of ferritin in an autophagy-independent manner^19^. The membrane protein ferroportin (FPN1) and Prominin2 (PROM2) establish the iron export pathways discovered thus far that maintain intracellular and plasma iron homeostasis in the presence of excess labile iron, endowing cancer cells with resistance to ferroptosis^20^, ^21^. Taken together, these data indicate that iron metabolism plays an essential role in ferroptosis governance through multiple mechanisms.

Cells are equipped with various defense mechanisms that prevent ferroptosis by stopping the lethal accumulation of lipid peroxides to counteract the increase in the degree of oxidative damage caused by metabolic activities associated with ferroptosis. Previous research has revealed three widely recognized defense mechanisms. First, the widely acknowledged canonical pathway has been shown to inhibit ferroptosis via the cystine/glutathione (GSH)/GPX4 signaling axis. System Xc^-^ mediates the internalization of extracellular cystine in conjunction with the export of intracellular glutamate (Glu), a process that is required to maintain reduced GSH levels^7^, ^22^. Stable GSH levels ensure the activity of glutathione peroxidase 4 (GPX4), which is characterized mainly by its unique enzymatic function in the reduction of phospholipid hydroperoxides^7^, ^23^. The classical ferroptosis inhibitor erastin and RSL3 block system Xc^-^ and GPX4 activities, respectively^22^, ^23^. Second, ferroptosis suppressor protein 1 (FSP1), previously known as apoptosis-inducing factor mitochondria-associated 2 (AIFM2), has been identified as a potent ferroptosis resistance factor. Mechanistically, FSP1 functions as an oxidoreductase to reduce coenzyme Q10 (CoQ or ubiquinone). In its fully reduced form, CoQH_2_ functions as a lipophilic free radical that traps the antioxidants that prevent the production of lipid peroxides^24^, ^25^, ^26^, ^27^. Third, ferroptosis in tumors with a specific genetic background is differentially regulated by sex hormones. Mechanistically, sex hormone signaling mediates the transcriptional upregulation of MBOAT1/2 to suppress ferroptosis in cancer cells, which selectively transfers MUFAs into lyso-PE, hence competitively reducing the PE-PUFA content^28^. Another study proposed that androgen receptor (AR) activates GPX4 expression in LAR tumors by directly binding to AR response elements located in the GPX4 promoter region^29^. Thus, AR lies at the nexus between PUFA metabolism and the GSH system. These novel findings underscore the concept that an integrated antioxidant system can effectively attenuate ferroptosis caused by excessive oxidative stress.

Ferroptosis ultimately depends on the catalytic oxidation of polyunsaturated fatty acids (PUFAs) in membrane phospholipids, resulting in the accumulation of lipid peroxides. PUFAs carry more than one unsaturated bond, which makes them vulnerable to the free radical attacks that induce lipid peroxidation. A recent study has revealed that PL-PUFA_2_, rather than PL-PUFA_1_, play a key role in the execution of ferroptosis. A selective reduction in PC-PUFA_2_ synthesis by MUFAs reverses the effect of free PUFAs, and thus MUFA supplementation prevents ferroptosis^30^. PUFA biosynthesis depends on certain lipid metabolic enzymes, such as acyl-CoA synthetase long-chain family member 4 (ACSL4) and lysophosphatidylcholine acyltransferase 3 (LPCAT3). The oxidation of PUFAs is mediated mainly by enzymatic reactions and spontaneous nonenzymatic autooxidation. The mitochondrial electron transport chain (ETC), nicotinamide adenine dinucleotide phosphate (NADPH) oxidase (NOX), and myeloperoxidase (MPO) are the sources of some of the ROS needed for ferroptosis^31^, ^32^, ^33^. Nonenzymatic lipid peroxidation occurs through the Fenton reaction, which is a catalytic process in which Fe^2+^ reacts with H_2_O_2_ to produce Fe^3+^, HO·, and OH-, resulting in free radical ions that cause oxidative damage to various cellular components, including membrane lipids^34^. However, the enzymatic oxidation of PUFAs and subsequent ferroptosis are mediated in a cell type-dependent manner by different members of the ALOX family, such as ALOX12 and ALOX15^35^, ^36^. Alternatively, POR drives lipid peroxidation in an ALOX-independent manner, promoting ferroptosis across a wide spectrum of lineages and cell states^37^. Therefore, the suggestion that iron chelation or inactivation of ACSL4, LPCAT3, or POR blocks or considerably attenuates ferroptosis is reasonable.

## 2. Mitochondrial quality control and ferroptosis

One of the morphological features that distinguishes ferroptosis from other forms of cell death is the notable change in the mitochondrial ultrastructure during ferroptosis. Engineered HRAS-mutant BJeLR tumor cells treated with erastin presented morphological characteristics that included a smaller organelle size, condensed mitochondrial membranes, which increased their density, and a reduction in the number of mitochondrial cristae; however, these cells exhibited none of the characteristic morphologic features associated with necrosis (e.g., cytoplasmic and organelle swelling, and plasma membrane rupture), apoptosis (e.g., chromatin condensation and margination), or autophagy (e.g., the formation of double-membrane entrapment vesicles)^1^.

**Figure 1:**
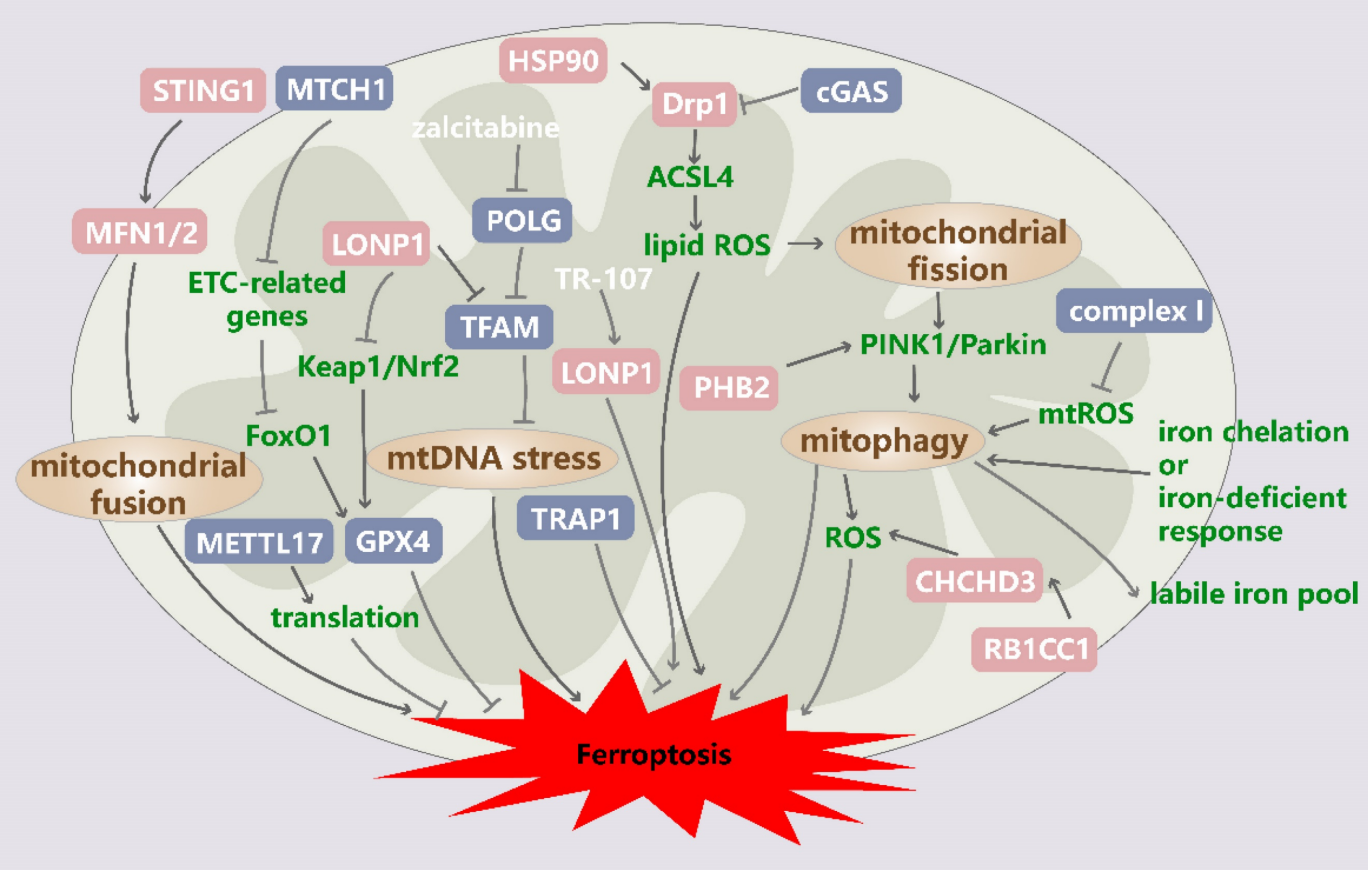
** Mitochondrial quality control in ferroptosis.** Zalcitabine targets POLG to induce the LONP1-dependent degradation of TFAM, thereby inducing mtDNA stress and subsequent ferroptosis. LONP1 is also involved in regulating ferroptosis mediated partly through the Keap1/Nrf2/GPX4 pathway. The RB1CC1-mediated upregulation of CHCHD3 stimulates MMP hyperpolarization and mtROS production to sensitize tumor cells to ferroptosis. TRAP1 plays a protective role in ferroptosis. STING1-MFN1/2 signaling triggers ferroptosis by inducing mitochondrial fusion. The upregulation of the HSP90/Drp1/ACSL4 pathway positively regulates ferroptosis via lipid ROS generation and altered mitochondrial morphology. MTCH1 deficiency downregulates mitochondrial ETC-related genes and promotes ferroptosis by triggering FoxO1-GPX4-axon-mediated retrograde signaling. METTL17 inhibition results in the impaired translation of mitochondrial protein-coding genes and increases ferroptosis sensitivity. Mitochondria-localized cGAS enhances DRP1 oligomerization to suppress ferroptosis. Mitochondrial fission is induced by increased ROS production, which drives PTEN-induced kinase 1 (PINK1)/Parkin-dependent mitophagy and ferroptosis. In addition, mitophagy is triggered by iron chelation or a cellular response to iron deficiency, and mitophagy tends to replenish the iron pool, ultimately leading to lipid peroxidation and ferroptosis. POLG: DNA polymerase gamma catalytic subunit; LONP1: Lon peptidase 1; TFAM: transcription factor A; Keap1: Kelch-like ECH-associated protein 1; Nrf2: NFE2-related factor 2; GPX4: glutathione peroxidase 4; RB1CC1: RB1-inducible coiled-coil 1; CHCHD3: coiled-coil-helix-coiled-coil-helix domain containing 3; TRAP1: tumor necrosis factor receptor-associated protein 1; STING1: stimulator of interferon response cGAMP interactor 1; MFN1/2: mitoferrin 1/2; HSP90: heat shock protein 90; Drp1: dynamin-related protein 1; ACSL4: acyl-CoA synthetase long-chain family member 4; MTCH1: mitochondrial carrier 1; PHB2: prohibitin 2.

Mitochondria have evolved multiple systems of quality control to ensure that the requisite number of functional mitochondria are available to meet the demands of cells^38^. Multilevel and well-orchestrated networks are involved in the maintenance of mitochondrial proteostasis, mitochondrial dynamics, mitophagy, and mitochondrial biogenesis (Figure 1).

### 2.1 Mitochondrial proteostasis

The mitochondrion-specific protein quality control system consists of chaperones and proteases that are critical for mitochondrial protein folding, which prevents the aggregation of misfolded proteins. Under overwhelming stress conditions, the mitochondrial unfolded protein response is activated to increase the expression of proteins involved in restoring mitochondrial proteostasis^39^. Previously, researchers had identified functional crosstalk between LONP1 and ClpP, which are two mitochondrial matrix proteases that cooperate to attenuate proteotoxic stress and protect mitochondrial functions to promote cancer cell survival^40^. The protein expression of both these factors is upregulated during ferroptosis and is accompanied by elevated mitochondrial ROS (mtROS) levels 41, 42. Inhibition of LONP1 expression induces the activation of the Nrf2/Keap1 signaling pathway and GPX4 expression, and the ability of LONP1 to mediate the degradation of substrates involved in mitochondrial biogenesis induce mitochondrial DNA (mtDNA) stress and ferroptosis in human pancreatic ductal adenocarcinoma (PDAC) cells^43^, ^44^. These observed functions differ from the expected role of LONP1 in protecting cells from ferroptosis. Similarly, inhibiting ClpP in leukemic cells leads to abnormal protein accumulation and causes cell death^45^, while the excessive activation of ClpP results in ETC degradation, which synergistically enhances the ferroptosis of leukemia cells in conjunction with GPX4 inhibition^46^. These results can be attributed to several reasons. LONP1 and ClpP are coexpressed at high levels in most cancers and share many target substrates, including proteins involved in oxidative phosphorylation, the TCA cycle, and amino acid and lipid metabolism^46^. These two proteases cooperate to maintain protein quality and target substrate activity to protect mitochondrial function, and their hyperactivation may lead to the excessive degradation of key mitochondrial components, ultimately activating the mitochondria-associated ferroptosis pathway and leading to irreversible cellular damage^40^, ^43^, ^44^. The therapeutic drug TR-107, a newly developed selective activator of ClpP, has shown encouraging preclinical efficacy against multiple malignancies. Mechanistically, TR-107 decreases the expression of proteins involved in the mitochondrial unfolded protein response and mitochondrial DNA transcription and translation, disrupts the mitochondrial respiratory chain, and upregulates mitophagy and ferroptosis^47^. In addition, tumor necrosis factor receptor-associated protein 1 (TRAP1), a member of the heat shock protein 90 (HSP90) chaperone family that resides mainly in mitochondria, has been shown to play a modest protective role in ferroptosis in melanoma cells^48^. Mechanistically, TRAP1 overexpression inhibits mPTP opening and increased ROS production, thereby preventing BAY-induced ferroptosis, while endogenous TRAP1 levels or TRAP1 activity are too low to prevent this outcome. However, in glioma, HSP90 binds to calcineurin and stimulates its activity, promotes the dephosphorylation of DRP1 at SER637, further binds to and stabilizes ACSL4, and ultimately sensitizes cells to ferroptosis^49^. Thus, the effects of HSP90 might depend on the context and molecular pathway.

In summary, the maintenance of mitochondrial proteostasis protects cells from ferroptosis-related oxidative stress, but highly active proteases may induce ferroptosis. Further studies are needed to fully understand the exact mechanisms regulating mitochondrial quality control and their interconnections among proteostasis modules to develop effective cancer therapeutic strategies.

### 2.2 Mitochondrial dynamics

Mitochondrial fission relies on the interaction between the cytoplasmic proteins dynamin-related protein 1 (DRP1) and fission 1 (Fis1), which form a circular structure that surrounds and contracts a site on the outer membrane of mitochondria, ultimately producing smaller spherical mitochondria^50^. The regulation of this process is closely linked to ferroptosis. DRP1 knockdown triggers mitochondrial filamentation, attenuates the impaired MMP and reduces basal and maximal mitochondrial respiration, thus exerting protective effects on ferroptosis by preserving mitochondrial integrity and maintaining redox homeostasis^48^. The upregulation of the HSP90-DRP1-ACSL4 pathway positively regulates ferroptosis in glioma via lipid ROS generation and mitochondrial morphology alteration^49^. Mitochondrial fission is induced by increased ROS production and mitochondrial membrane potential abrogation, driving PTEN-induced kinase 1 (PINK1)/Parkin-dependent mitophagy and ferroptosis in non-small-cell lung cancer (NSCLC) cells, thus enhancing the anticancer effect of celastrol^51^. A consistent phenomenon was also observed in AML cells and malignant mesothelioma^52^, ^53^. In summary, increased ROS production tends to induce mitochondrial fission, which may induce mitophagy and ferroptosis. cGAS has been recognized as a cytosolic DNA sensor that activates innate immune responses through the production of the second messenger cGAMP and the adaptor protein STING. Recently, Qiu *et al.* reported the surprising finding that cGAS contains a mitochondrial targeting sequence (MTS) and is translocated to the OMM. Additionally, mitochondria-localized cGAS promotes cancer progression by suppressing ferroptosis in a manner dependent on an increase in DRP1 oligomerization instead of the canonical cGAS-STING pathway^54^. Notably, DRP1 is regulated by posttranslational modifications (such as ubiquitination and phosphorylation) and stress to function in mitochondria^49^, ^51^, ^55^, ^56^. Therefore, targeting a single gene to regulate ferroptosis through mitochondrial fission may provide new insights for cancer treatment.

Mitochondrial fusion is a cellular response that reduces mitochondrial stress induced by aging or damaged proteins and ROS; it allows damaged mitochondria to undergo functional repair and ameliorates the detrimental effects of heteroplasmic mtDNA mutations^57^. The coordinated fusion of inner and outer membranes is mainly driven by optic atrophy 1 (OPA1) and mitofusins (including MFN1 and MFN2). Because of these outcomes, mitochondrial fusion seems to relieve oxidative damage and protect cells from ferroptosis. Several lines of evidence suggest that defective mitochondrial quality control contributes to heightened mitochondrial fission, reduced mitochondrial fusion, and dysfunctional mitophagy, resulting in decreases in mitochondrial biogenesis and mitochondria pathway-mediated cell death^55^, ^56^, ^58^, ^59^, ^60^, ^61^, ^62^, ^63^, ^64^, ^65^. However, a study provided evidence showing that STING1/MFN1/2-mediated mitochondrial fusion promotes mitochondrial oxidative damage and subsequent ferroptosis in PANC1 cells independent of the mitochondrial DNA damage response and PINK1-dependent mitophagy^66^. This outcome may have been a result of multiple effects of mitochondrial dynamics, which regulate more processes than quality control; for example, mitochondrial dynamics affect systems involved in nutrient utilization and energy expenditure^67^. Excessive mitochondrial fission may destroy OXPHOS process, while increased mitochondrial fusion may generate mtROS during OXPHOS or increase the conversion of fatty acids to acetyl-CoA, leading to lipid peroxidation and ferroptosis^66^. Additionally, OPA1 has been reported to promote ferroptosis in osteosarcoma independent of mitochondrial fusion. Mechanistically, OPA1 deficiency promotes ferroptosis resistance by attenuating mtROS production and inducing the activating transcription factor 4 (ATF4)-dependent upregulation of the xCT-GSH-GPX4 pathway as part of an integrated stress response. This outcome highlights the role of OPA1 in modulating the mitochondrial structure and energetics (respiratory supercomplex assembly, cristae remodeling, and oxidative phosphorylation), in addition to mitochondrial fusion^68^.

Therefore, mitochondrial fission and fusion are both reported to exert effects on ferroptosis.

### 2.3 Mitophagy

Mitophagy has been shown to facilitate tumorigenesis and the survival of various cancer cells by removing dysfunctional mitochondria^69^. It plays a pivotal role in reinstating cellular homeostasis under normal physiological and stress conditions by limiting mtROS production. Based on the role of mitophagy in response to increased mtROS production, a reasonable assumption is that mitophagy exerts a protective effect on ferroptosis. The therapeutic drug sappanone A has been reported to induce ferroptosis in NSCLC by regulating mitophagy and mitochondrial biogenesis. Mechanistically, sappanone A significantly inhibits PINK1/Parkin- and FUNDC1-mediated mitophagy, thereby disrupting downstream Nrf2-mediated mitochondrial energy metabolism (decreased ATP synthesis and mitochondrial respiratory function, as well as mPTP dysfunction) and mitochondrial biogenesis, ultimately activating ferroptosis mediated by the mitochondrial pathway^58^. However, increases in mitophagy and associated ROS production have been reported to lead to ferroptosis in melanoma cells and pancreatic cancer cells^48^, ^53^. These data suggest that enhanced mitophagy is a typical early response that promotes survival, while overwhelming or sustained mitochondrial damage can induce the pathological activation and acceleration of mitophagy, which leads to elevated mtROS production through a positive feedback loop, thus inducing ferroptosis^70^. As shown in a study by Basit F *et al.*, mitochondrial complex I inhibition increases mtROS levels, stimulates mitophagy, and triggers an increase in mitophagy-dependent ROS production, ultimately leading to ferroptosis^48^.

In terms of the mechanism, recent studies have focused mainly on the PINK1/Parkin pathway. PINK1 recruits Parkin, an E3 ubiquitin ligase, from the cytoplasm to mitochondria with a low membrane potential to initiate the autophagic degradation of damaged mitochondria^71^. In NSCLC cells, PINK1 knockdown reduced ubiquitinated protein levels in mitochondria, inhibited mitophagy and prevented ferroptosis^51^. In addition, prohibitin 2 (PHB2), a conserved inner mitochondria membrane (IMM) protein with functions in development, lifespan regulation, and diverse mitochondrial processes, and has been identified as a crucial mitophagy-related receptor protein during Parkin-mediated mitophagy in mammalian cells^51^. In mtDNA-depleted 143B human osteosarcoma (ρ^0^) cells, PHB2 expression was significantly downregulated after H_2_O_2_ treatment, contributing to an elevation in aquaporin expression and an associated increase in mitochondrial membrane permeability, thereby increasing cellular sensitivity to ferroptosis^72^. In addition to ubiquitin-dependent mitophagy, receptor-mediated mitophagy also plays a role in ferroptosis-related oxidative stress. For example, BNIP3- and NIX-mediated mitophagy defects increase mtROS levels, which synergistically increase the sensitivity of HeLa cells to ferroptosis with impaired Nrf2-driven antioxidant enzyme systems^73^. FUNDC1-mediated mitophagy plays a similar role in protecting against ferroptosis^58^. In addition, PINK1/Parkin-independent mitophagy has been reported to cause ferroptosis^53^, ^74^. Following CA9 inhibition in a hypoxic environment, malignant mesothelioma cells exhibit an iron-deficient response via accelerated iron intake processes and low stored iron levels, ultimately leading to lipid peroxidation and ferroptosis^53^. Mitophagy may be involved in the increase in the labile iron pool to some extent by releasing iron from numerous iron-sulfur clusters (ISCs) involved in oxidative phosphorylation^53^. Another study showed that iron chelation led to mitophagy independent of PINK/Parkin pathway activity. This induction of mitophagy may not be related to the damage response, but may recycle iron to maintain other essential iron-dependent functions^75^.

In summary, mitophagy may function during ferroptosis by regulating energy metabolism or increasing the levels of metabolic substrates.

### 2.4 Mitochondrial biogenesis

Human mitochondrial DNA encodes a portion of the ETC components involved in the oxidative phosphorylation needed for ATP synthesis. mtDNA integrity is critical for maintaining the cellular energy supply and cell cycle and for programmed cell death.

Multiple transcription factors and coactivators have been shown to orchestrate nuclear and mitochondrial gene expression during mitochondrial biogenesis. Nrf2 was the first nuclear transcription factor shown to be involved in the transcription of several mitochondrial genes encoding subunits of mitochondrial respiratory chain complexes and heme biosynthetic enzymes that localize to the mitochondrial matrix^76^. A study has shown that the upregulated Nrf2-dependent transcription of heme oxygenase-1 (HO-1) promotes ferroptosis in colorectal cancer (CRC) cells^77^. However, another study showed that the inhibition of the Nrf2/HO-1 axis promotes ferroptosis in KRAS-mutant CRC cells^78^. These inconsistent results suggest that the involvement of Nrf2 in mitochondrial biogenesis plays a context-dependent role in ferroptosis. In fact, Nrf2 is a key hub gene involved in the regulation of ferroptosis, orchestrating multiple effects on mitochondrial biogenesis, mitochondrial fission and fusion, and mitophagy^79^. Mitochondrial transcription factor A (TFAM) stabilizes mtDNA and regulates the amount of mtDNA by forming nucleoids^80^. The degradation of TFAM has been shown to induce mtDNA stress and macroautophagy/autophagy-dependent ferroptosis in human pancreatic cancer cells^44^. In addition, Huang C, *et al.* found that TFAM complementation completely reversed mitochondrial network disorganization, oxidative DNA damage and ferroptosis induced by the impaired ability of PDAC- and HCC-derived cells to respond to stress^81^. In conclusion, TFAM, a key regulator of mitochondrial biogenesis, can be considered an antagonist of ferroptosis.

Researchers found that cytochrome c oxidase subunit 7a (COX7A1), an important complex in the mitochondrial ETC, increased the sensitivity of NSCLC cells to CDI ferroptosis, accompanied by the downregulation of PINK, Parkin, PGC-1α (mitochondrial biogenesis activator), TFAM, DRP1, and MFN1, indicating its inhibition of mitochondrial biogenesis, mitophagy, and mitochondrial fusion and fission^82^. This result may be explained by the fact that these mitochondrial processes occur dynamically, with one mechanism dominating after counteracting another. As part of the mitochondrial inner membrane structure regulatory complex, coiled-coil-helix-coiled-coil-helix domain containing 3 (CHCHD3) plays an important role in increasing the stability and cristae morphology of mitochondria. Xue, Xiangfei *et al.* reported that RB1-inducible coiled-coil 1 (RB1CC1)-mediated upregulation of CHCHD3 stimulates MMP hyperpolarization and mtROS production to sensitize tumor cells to ferroptosis^83^. Mitochondrial carrier 1 (MTCH1) is a mitochondrial outer membrane protein insertase. Its deficiency downregulates mitochondrial ETC-related genes, thereby disrupting mitochondrial OXPHOS and governing ferroptosis by triggering FoxO1-GPX4-axon-mediated retrograde signaling^84^. These results highlight the important roles of the abnormal expression and regulation of genes involved in maintaining mitochondrial integrity and respiratory function in the process of ferroptosis.

Epigenetic modulation also plays an important role in controlling mitochondrial function. Li H, *et al.* found that the inhibition of the mitochondrial protein METTL17 or its interacting proteins significantly reduced mitochondrial RNA methylation, leading to the impaired translation of mitochondrial protein-coding genes and increased ferroptosis sensitivity^85^. However, ρ^0^ cells displayed the same sensitivity to ferroptosis inducers as parental cells^1^. This outcome may be attributable to a compensatory response to the prolonged lack of mitochondria in which the metabolism in these cells is fundamentally rewired, and thus, mitochondrion-independent ferroptosis is regulated by a mechanism that differs from that of the parental cells^13^.

Despite some inconsistent results, the modulation of mitochondrial biogenesis can be concluded to be closely related to ferroptosis. Further in-depth investigations of the mechanisms of the interactions among these processes of mitochondrial dynamics and ferroptosis may lead to new ideas for the prevention and treatment of cancer.

## 3. Mitochondrial metabolism in ferroptosis

### 3.1 Tricarboxylic acid (TCA) cycle regulation in ferroptosis

The TCA cycle is a central metabolic pathway in the mitochondrial matrix, and it is a ubiquitous cycle in aerobic organisms and the nexus of the glucose, lipid, and amino acid metabolic pathways in the body. In the TCA cycle, acetyl-CoA derived from carbohydrates, fatty acids, amino acids, and ketone bodies is oxidized, and NADH and FADH_2_ are produced for use in the ETC; eight enzymes are involved in the TCA cycle^86^. The mitochondrial protein pyruvate dehydrogenase kinase 4 (PDK4) functions as a ferroptosis inhibitor in an acetyl CoA carboxylase (ACC)-dependent manner, blocking pyruvate dehydrogenase-dependent pyruvate oxidation and subsequent citric acid and fatty acid production in PDAC cells to ultimately inhibit glucose-dependent ferroptosis^87^. In contrast, although pyruvate also enters the TCA cycle through pyruvate carboxylase (PC)-mediated oxaloacetate production, PC knockdown failed to downregulate ferroptosis^87^. Thus, the maintenance of the TCA cycle may contribute to ferroptosis by switching the glucose metabolism machinery to increase fatty acid synthesis. Mitochondrial NADP+-dependent isocitrate dehydrogenase (IDH2) catalyzes the conversion of isocitrate to α-ketoglutarate (α-KG), the first oxidative decarboxylation reaction in the TCA cycle. The downregulation of IDH2 sensitizes cancer cells to ferroptosis by reducing the mitochondrial NADPH pool, which is a major site of mitochondrial GSH turnover^88^. α-Ketoglutarate dehydrogenase (α-KGDH) converts α-KG to succinyl-CoA, and when its E3 component DLD is silenced, lipid peroxidation and cystine deprivation-induced ferroptosis in head and neck cancer cells is inhibited via a mechanism involving glutaminolysis^89^. Glutamine (Gln) is a nonessential amino acid and major fuel source for energy production and lipid biosynthesis for respiration mediated by glutaminolysis in proliferating cells. Tumor cells exhibit a Gln dependence that is rarely seen in normal cells because glutaminolysis replenishes the number of TCA cycle intermediates and supports the biosynthetic processes required for tumor growth^90^. Thus, glutaminolysis has been implicated in ferroptosis regulation in tumor cells^13^, ^91^. Cystine deprivation failed to induce lipid ROS accumulation or ferroptosis in the absence of Gln or after glutaminolysis blockade, and this outcome was offset by supplementation with α-KG and its downstream metabolites, such as succinate, fumarate, and malate^13^, ^91^. Previous studies revealed that ferroptosis was abolished by blocking the production of components involved in a series of reactions involved glutaminolysis; these components included SLC1A5 and SLC38A1 (involved in Gln intake), GLS2 (converts Gln to Glu), and transaminase and GLUD1 (converts Glu to α-KG)^91^, ^92^. Additionally, ASS1, a key enzyme participating in the urea cycle, promotes the reductive carboxylation of Gln, blocks Gln from entering the TCA cycle, reduces mitochondrial lipid ROS production, and promotes de novo MUFA synthesis through a mechanism dependent on reductive pathway-derived acetyl-CoA, thereby conferring ferroptosis resistance to cancer cells^93^. These data highlight that the anaplerotic reactions mediated by glutaminolysis are indispensable for cysteine deprivation-induced (CDI) ferroptosis. In addition, mitochondrial pyruvate carrier 1 (MPC1) in the IMM internalizes pyruvate into mitochondria. MPC1 suppression increases the vulnerability of cancer cells to ferroptosis, which retained their mesenchymal cell traits and maintained glutaminolysis^94^. Taken together, two distinct upstream pathways (pyruvate oxidation and Gln anaplerosis) may increase ferroptosis sensitivity via their TCA cycle-related activities, through which they not only mediate ROS production but also promote fatty acid synthesis^87^.

In another biological role, the TCA cycle provides energy for the body. Through acetyl-CoA, which the initiates the cycle, electrons are transferred to the ETC, promoting the production of ATP through oxidative phosphorylation. AMP-activated protein kinase (AMPK), an energy sensor whose activity is modulated based on the cellular ADP:ATP ratio, plays a dual role in regulating ferroptosis via its phosphorylation of substrates. For example, glucose starvation prevents ferroptosis in mouse embryonic fibroblasts (MEFs) by activating AMPK-mediated phosphorylation of acetyl-CoA^95^. In contrast, AMPK-mediated phosphorylation of beclin 1 (BECN1) plays an autophagy-independent role in promoting ferroptosis by directly blocking system Xc^-^ activity in CRC cells^96^. Additionally, AMPKα1/α2 knockdown in PANC1 cells or MEFs leads to distinct cellular sensitivity to erastin- or RSL3-induced death under high-glucose or glucose starvation conditions, indicating that the role of AMPK in ferroptosis is cell type-dependent and that AMPK is involved in the complex relationship between ferroptosis and the cellular energy state^87^. Recently, research revealed that activated AMPK may inhibit ferroptosis by regulating pyrimidinosome formation. Mechanistically, stress-activated AMPK dissociates from the pyrimidinosome to increase pyrimidine flux up to the biosynthesis of DHOA/OA. Then, DHOA can be safely secreted from cells to relieve reductive stress or converted to OA by DHODH^97^, ^98^. However, AMPK activators and DHODH inhibitors can synergistically induce ferroptosis in cancer cells; thus, the active AMPK-promoted pyrimidinosome-mediated ferroptosis defense appears to serve as a protective pathway to prevent self-jury, and the DHODH-mediated metabolic conversion potentially plays a key role in antagonizing AMPK-related stresses^99^. The TCA cycle supports the electron transport activity of protein complexes located in the IMM. As shown by Gao *et al.*, the inhibition of ETC mitochondrial complexes I, II, III, and IV inhibited ROS accumulation and ferroptosis induced by cystine starvation and erastin activity in HT-1080 cells, but the GPX4 inhibitor RSL3 had no effect^13^. The different outcomes may be related to the subcellular localization of erastin and RSL3; notably, erastin has been shown to target voltage-dependent anion-selective channels (VDACs) in mitochondria, while GPX4 is the most downstream component in the defensive antiferroptotic pathway and is critical for lipid ROS clearance.

**Figure 2:**
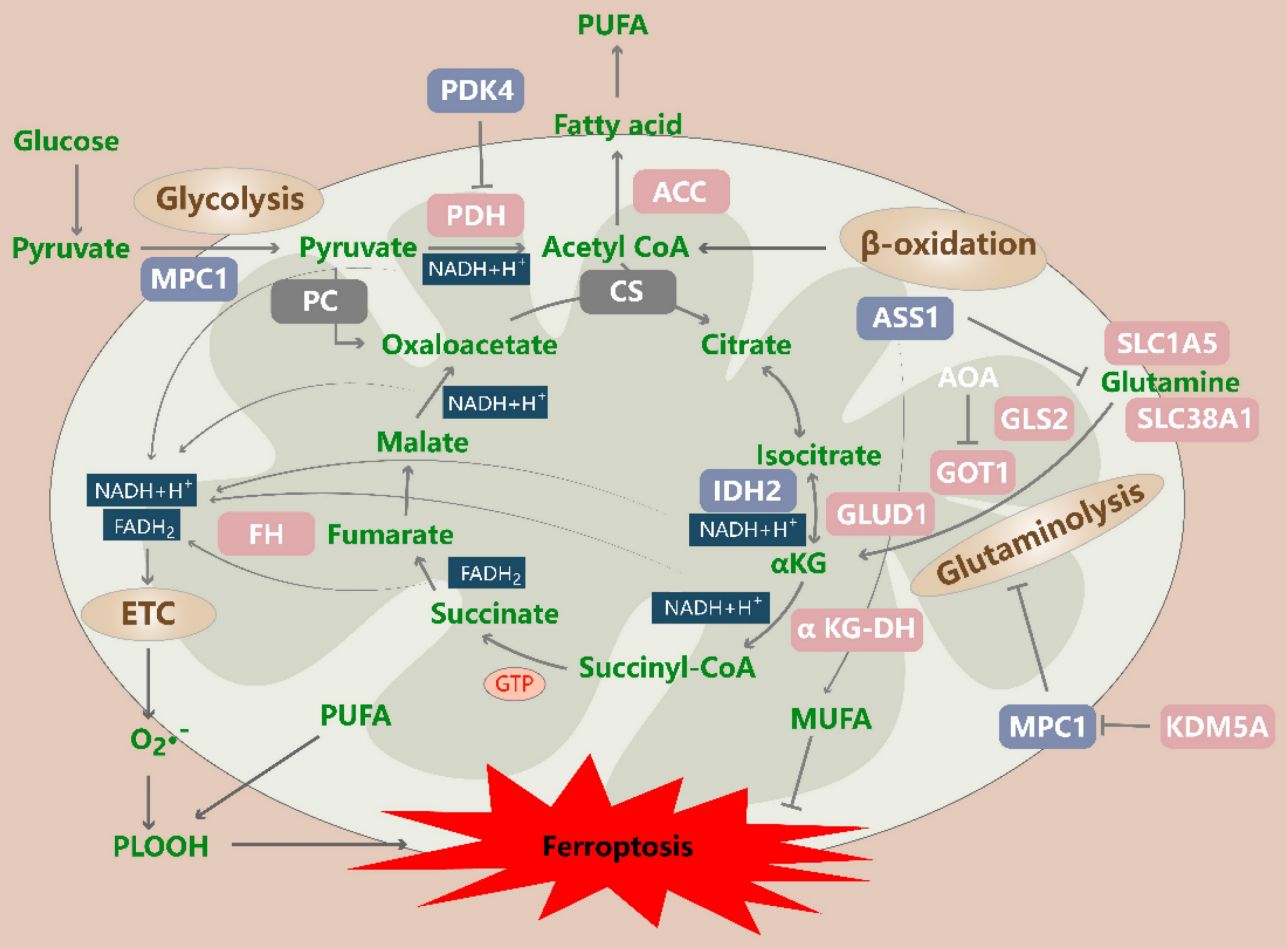
** Regulation of the tricarboxylic acid (TCA) cycle in ferroptotic cells.** The TCA cycle drives ferroptosis by transferring electrons to the electron transport chain (ETC), which generates ROS, as well as by promoting ACC-mediated fatty acid synthesis. Glycolysis, β-oxidation, and glutaminolysis produce TCA cycle precursors, and this production is suppressed by PDK4-mediated glucose metabolism and increased by enhanced glutaminolysis and exogenous supplementation with TCA cycle substrates. In addition, MPC1 inhibits ferroptosis in part by inhibiting glycolysis and glutaminolysis. ACC: acetyl-CoA carboxylase; PDK4: pyruvate dehydrogenase kinase 4; MPC1: mitochondrial pyruvate carrier 1; PDH: pyruvate dehydrogenase; PLOOH: phospholipid hydroperoxide; PUFA: polyunsaturated fatty acid; PC: pyruvate carboxylase; CS: citrate synthase; GLS2: glutaminase 2; GOT1: glutamic-oxaloacetic transaminase 1; GLUD1: glutamate dehydrogenase 1; IDH2: isocitrate dehydrogenase (NADP[+]) 2; FH: fumarate hydratase; α-KGDH: alpha-ketoglutarate dehydrogenase subunit E2; KDM5A: lysine demethylase 5A; ETC: electron transport chain; SLC1A5: solute carrier family 1 member 5; SLC38A1: solute carrier family 38 member 1.

In summary, the TCA cycle is involved in the regulation of ferroptosis through its role as a major metabolic hub and its function in altering the energy status (Figure 2).

### 3.2 Association of mitochondrial ROS production with ferroptosis

ROS are produced during oxidative stress; these ROS include superoxide anions (O_2·_^-^), hydrogen peroxides (H_2_O_2_), hydroxyl radicals (HO·), lipid hydroperoxides, and peroxy radicals, which mediate intracellular signal transduction. They are mainly produced by NOX and the ETC in endogenous biological systems^32^, ^100^.

**Figure 3:**
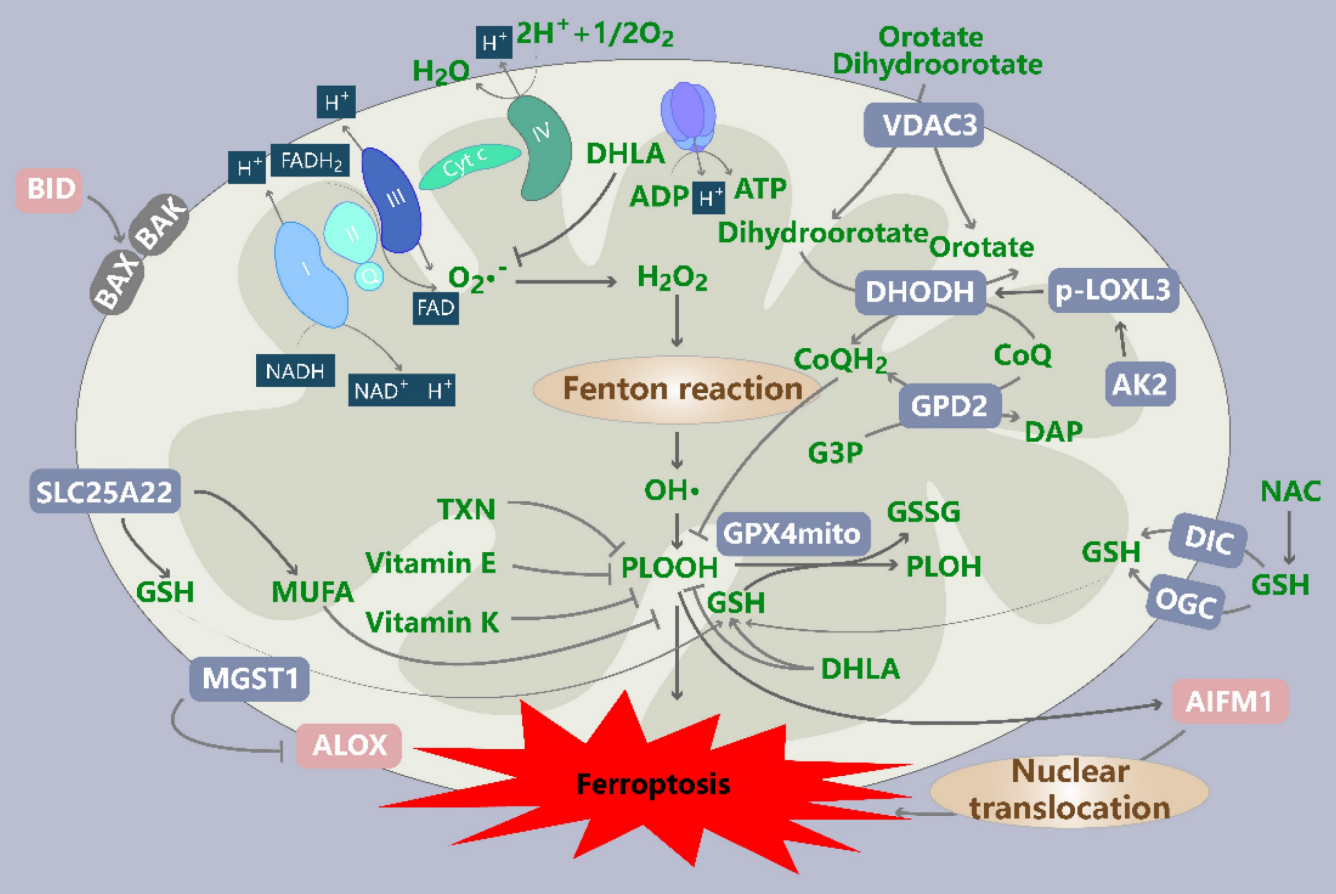
** Association of mitochondrial ROS production with ferroptosis.** GPX4, DHODH, GPD2, AK2 and MGST1 are mitochondrial antioxidant enzymes that play important roles in protecting cells from ferroptosis. Nonenzymatic substances also play pivotal roles. OMM-localized VDAC3 serves as a ferroptosis suppressor and provides internal access for DHOA and OA. SLC25A22, a driver of ferroptosis resistance, mediates the production of GSH and MUFAs. DIC and OGC transport glutathione (GSH) into mitochondria, and their inhibition can lead to GSH depletion and an increased ferroptosis rate. TXN, vitamin E, vitamin K, dihydrolipoic acid (DHLA) and N-acetylcysteine (NAC) also possess antioxidant activities. In addition, the release of AIFM1 induced by mitochondrial oxidative damage promotes ferroptosis after its translocation to the nucleus. BID mediates mitochondrial dysfunction, mtROS production and ferroptosis. DHODH: dihydroorotate dehydrogenase; G3P: glycerol-3-phosphate; GPD2: G3P dehydrogenase 2; AK2: mitochondrial adenylate kinase 2; MGST1: microsomal glutathione S-transferase 1; ALOX: lipoxygenase; DIC/SLC25A10: solute carrier family 25 member 10; OGC/SLC25A11: solute carrier family 25 member 11; TXN: thioredoxin; DHLA: dihydrolipoic acid; NAC: N-acetylcysteine; AIFM1/AIF: apoptosis-inducing factor mitochondria-associated 1; BID: BH3-interacting domain death agonist.

An increase in the levels of mitochondrial ROS facilitates ferroptosis, which can be inhibited by mitochondrion-targeted antioxidants (Figure 3).

#### 3.2.1 Mitochondrion-targeted ROS scavengers

First, mitochondrial lipid ROS levels are increased significantly and induce ferroptosis in erastin-treated HT-1080 cells and MEFs, as measured by the C11 BODIPY 581/591 staining intensity and the quantitative analysis of malondialdehyde (MDA), mitoPeDPP and MitoSOX levels^13^. An imbalance between oxidative stress levels and antioxidant system activity drives ferroptosis. Therefore, mitochondrion-targeted ROS scavengers such as mitoTEMPO, mitoquinone (MitoQ), and XJB-5-131 function as exogenous antioxidant mechanisms in oxidative stress resistance and reverse ferroptosis in a variety of cancer cell types and animal models^98^, ^101^.

#### 3.2.2 Mitochondrial antioxidant enzymes

Second, several mitochondrial antioxidant enzymes play vital roles in inhibiting ferroptosis. GPX4^cyto^ is a pivotal component of the cellular antioxidant defense system, and studies have shown that the mitochondrial form GPX4^mito^ plays a key role in mitigating mitochondrial oxidative damage during ferroptosis^102^. DHODH mediates the oxidation of dihydroorotate to yield orotate, which is coupled with the reduction of CoQ to CoQH_2_. The combination of DHODH knockdown and GPX4 inactivation, but not either alone, substantially induced mitochondrial lipid peroxidation and ferroptosis in cancer cells, which was reversed by the restoration of GPX4^mito^ but not GPX4^cyto^^98^. These data suggest that DHODH is an antioxidant component that functions in parallel with GPX4^mito^ in mitochondria. Similar to DHODH deficiency, cell death induced by VDAC3 depletion is partially prevented by ferrostatin-1 and is more potent when combined with uridine supplementation, as the OMM-localized VDAC3 protein serves as a platform to integrate the pyrimidinosome and provide internal access for DHOA and OA^99^. In addition, AMPK agonists and DHODH inhibitors synergistically increase lipid ROS levels and induce ferroptosis in cancer cells, and low expression of the AMPK protein renders cancer cells more dependent on DHODH-mediated ferroptosis defenses^99^. Thus, DHODH serves as a hub connecting substance metabolism, energy metabolism, and defense systems. IMM-localized glycerol-3-phosphate (G3P) dehydrogenase 2 (GPD2) exerts an effect similar to that of DHODH in that it catalyzes G3P oxidation, which is coupled with the reduction of CoQ to CoQH_2_, which functions as a radical-trapping antioxidant in mitochondria that suppresses ferroptosis. The supplementation of cancer cells with G3P attenuates ferroptosis triggered by GPX4 inhibitors in a GPD2-dependent manner^103^. As a member of the lysyl-oxidase (LOX) family, LOXL3 can be transported to mitochondria in a manner dependent on OMM-localized TOM20. Researchers found that mitochondrial adenylate kinase 2 (AK2) phosphorylates LOXL3-S704 to enhance lysyl oxidase activity and block DHODH-K344 ubiquitination, in turn conferring resistance to chemotherapy-induced ferroptosis^104^. In addition, microsomal glutathione S-transferase 1 (MGST1), a membrane-bound transferase located mainly in mitochondria, the endoplasmic reticulum (ER), the plasma membrane, and peroxisomes that plays a clear role in the conjugation of electrophiles and oxidative stress protection, has also been reported to bind to ALOX5, limiting ALOX5-mediated lipid peroxidation and subsequent ferroptosis in an Nrf2-dependent manner in pancreatic cancer cells^105^. In conclusion, mitochondrial antioxidant enzymes play roles in defensive antiferroptotic responses by eliminating lipid peroxidation damage (directly or indirectly by producing free radical scavengers) or blocking the enzymatic oxidation of PUFA-phospholipids (PLs).

#### 3.2.3 Nonenzymatic mitochondrial antioxidants

Third, a variety of nonenzymatically generated substances are involved in the antioxidant system. As a core contributor to ferroptosis, GSH is not produced in mitochondria and cannot penetrate the IMM but is transported into mitochondria by potential carrier proteins, such as the dicarboxylate carrier (DIC, SLC25A10) and the oxoglutarate carrier (OGC, SLC25A11). SLC25A22 (a mitochondrial glutamate transporter)-dependent NAPDH synthesis mediates the production of GSH^106^. Low GSH levels in mitochondria can also reduce the S-glutathionylation rate of mitochondrial proteins, especially the ETC complexes and TCA cycle enzymes, and diminish their activity. This feedback loop further increases ROS generation and GSH consumption in mitochondria^107^. N-acetylcysteine (NAC) is the acetylated precursor of GSH and shows strong antioxidant activity. NAC has been reported to block the increases in ROS production and ferroptosis in hepatocellular cancer (HCC) and PDAC cells^81^, ^108^. In addition, biological antioxidants such as CoQ, lipoic acid and its reduced form dihydrolipoic acid (DHLA), thioredoxin (TXN), and vitamins E and K have all been reported to play protective roles in ferroptosis^109^, ^110^, ^111^, ^112^, ^113^. Notably, most of the major regulatory factors of the aforementioned endogenous antioxidant defense system are, at least in part, associated with Nrf2; these enzymes include GPX4^114^ and MGST1^115^, as well as vitamin E^116^. In summary, antioxidants such as vitamin E antagonize the Nrf2-Keap1 interaction and lead to the stabilization and activation of Nrf2^116^. Moreover, Nrf2 maintains redox homeostasis by modulating antioxidant-response element (ARE)-dependent transcription and the expression of antioxidant defense enzymes, playing vital roles in lipid peroxidation and ferroptosis prevention^114^, ^117^.

### 3.3 Mitochondrial iron metabolism and ferroptosis

**Figure 4:**
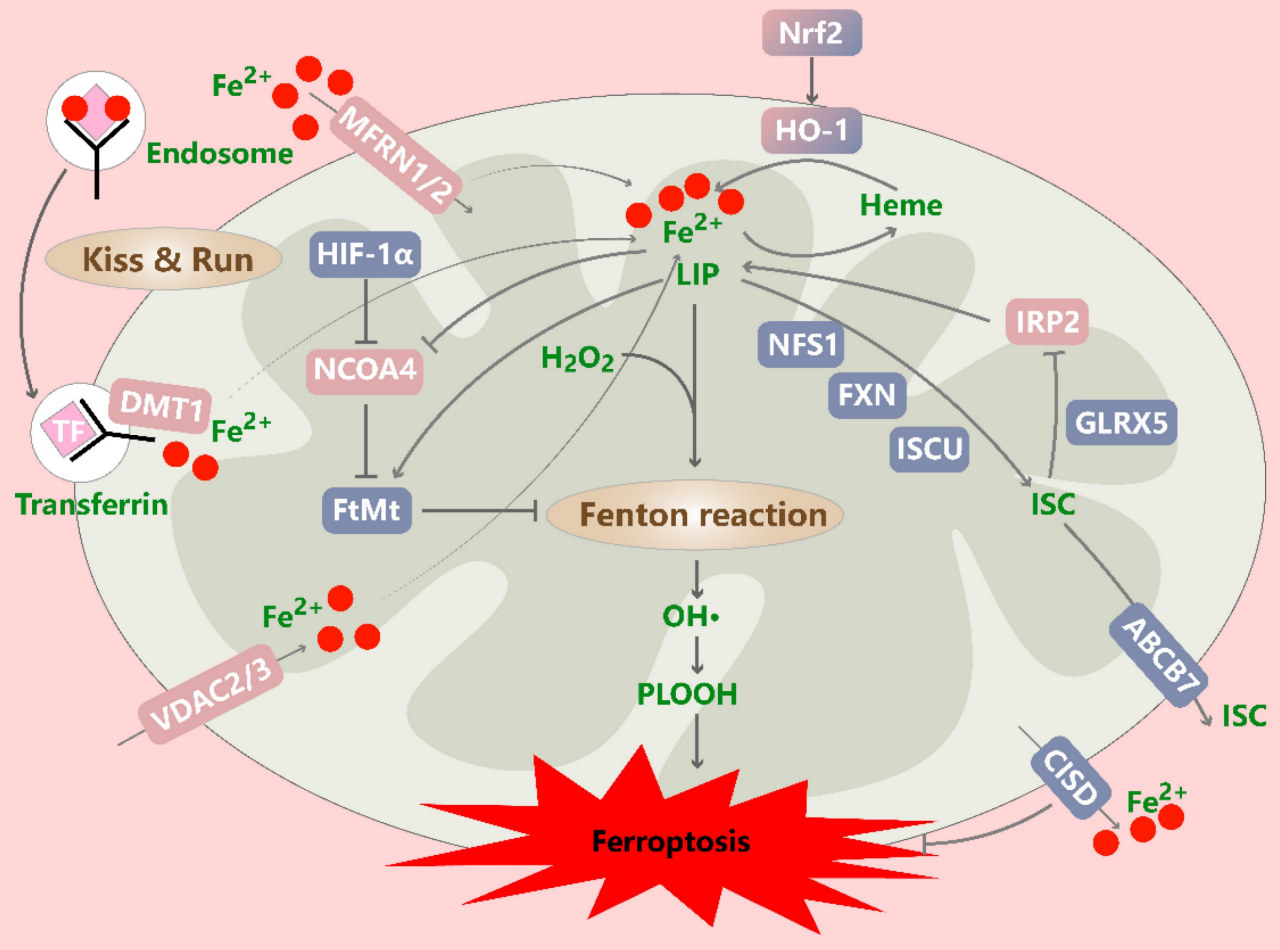
** Mitochondrial iron metabolism in ferroptotic cells.** Iron can be transported into mitochondria through MFRN1/2, VDAC2/3 and “kiss and run” pathways, is mainly used for the synthesis of heme and iron-sulfur clusters (ISCs), and is also stored in mitochondrial ferritin. Excess iron promotes ferroptosis through the Fenton reaction. The HO-1-mediated degradation of heme promotes Fe^2+^ release and regulates ferroptosis in a cell type-dependent manner. Interference with the biosynthesis (NFS1, FXN, and ISCU), transfer (GLRX5), and export (ABCB7) of ISCs enhances ferroptosis. In addition, FtMt levels can be regulated by HIF-1α and NCOA4. CISD mediates Fe^2+^ transport out of mitochondria, thereby inhibiting ferroptosis. MFRN1/2: mitoferrin 1/2; VDAC2/3: voltage-dependent anion-selective channel 2/3; HO-1: heme oxygenase-1; NFS1: cysteine desulfurase; FXN: frataxin; ISCU: iron-sulfur cluster assembly enzyme; GLRX5: glutaredoxin 5; FtMt: mitochondrial ferritin; NCOA4: nuclear receptor coactivator 4; CISD: CDGSH iron sulfur domain.

Mitochondria are not only hubs of cellular energy metabolism but also the main organelles involved in intracellular iron regulation (Figure 4). Iron must cross the outer mitochondrial membrane (OMM) and IMM to enter the mitochondrial matrix, where it is involved in various metabolic activities. Mitochondrial iron is involved mainly in the biosynthesis of heme and ISCs, and it is stored by mitochondrial ferritin (FtMt). The dependence of cancer cells on iron to maintain their rapid growth rate makes them more sensitive to the dysregulation of iron metabolism. During rapid cell proliferation, more iron may be imported into mitochondria to meet the increasing demand for cofactors^118^. However, excessive mitochondrial iron levels can induce ROS generation or abnormal iron-containing enzyme activity, promoting ferroptosis.

#### 3.3.1 Iron transport

Several molecules involved in mitochondrial iron transport have been found to contribute to ferroptosis. First, VDACs function as gatekeepers for the entry and exit of metabolites and ions between the cytosol and the mitochondria. Erastin treatment induces VDAC2/3 opening and is associated with mitochondrial iron accumulation and iron-dependent ferroptosis. Recent research has shown that VDAC3 ubiquitination increases the ferroptosis sensitivity of gastric cancer cells due to the loss of control over iron ion entry into mitochondria^119^. Second, iron transport across the IMM can be mediated by the membrane transporter mitoferrin 1 (Mfrn1) and its homolog mitoferrin 2 (Mfrn2), also known as the mitochondrial solute carrier family members SLC25A37 and SLC25A28, respectively^120^. *In vitro* and *in vivo* evidence suggest that Mfrn1 acts as a tumor suppressor to regulate mitochondrial iron-induced ferroptosis in HCC^121^. A previously study revealed the Mfrn2-dependent mitochondrial iron uptake induced iron-dependent mitochondrial dysfunction and the subsequent killing of human head and neck squamous carcinoma cells^122^. However, Mfrn1/2-mediated mitochondrial iron accumulation promotes osteosarcoma carcinogenesis, and high expression of Mfrn1 in GBM tumor tissues increases the level of mitochondrial iron and is associated with a poor prognosis^123^, ^124^. Overall, the expression and function of Mfrn1/2 may vary depending on the cell types and conditions^123^. Alternatively, mitochondria directly ingest iron through transient interactions with endosomes containing iron-bound transferrin (in a “kiss and run” process)^125^. Endosome docking is mediated by divalent metal transporter 1 (DMT1) located in the OMM, and DMT1 knockdown blocks erastin-induced ferroptosis by boosting GSH antioxidant defenses and mitophagy^126^. Another pathway apparently induces DMT1-independent iron uptake, but its characteristics remain to be elucidated^127^.

Previous localization studies showed that CDGSH iron sulfur domain 1 (CISD1, also called mitoNEET) is an integral protein in the OMM that has been reported to regulate VDAC1 activity in a redox-dependent manner, leading to pore closure and likely disrupting the flow of metabolites through VDACs^128^. The depletion of CISD1 drove iron-mediated intramitochondrial lipid peroxidation and ferroptosis in HCC cells^129^. In addition, given the role of VDAC1 in the regulation of mitophagy and the ability of CISD1 to mediate iron and ROS homeostasis in mitochondria, CISD1 has been hypothesized to regulate mitophagy-related ferroptosis by targeting VDAC1^130^, ^131^. CISD2 and CISD3 are both members of the NEET protein family and have been proposed to prevent ferroptosis in cancer cells^132^, ^133^, ^134^. Mechanistically, CISD2 inhibition leads to cellular autophagy, which has been implicated in the activation of ferritinophagy, resulting in an increased cellular labile iron pool^133^. Moreover, studies have shown that CISD3 depletion leads to metabolic reprogramming toward glutaminolysis, which is required for fueling mitochondrial oxidative phosphorylation, and that ferroptosis induced by CISD3 knockdown can be rescued by mitophagy, which eliminates damaged mitochondria^132^. In summary, NEET protein family members regulate ferroptosis through various mechanisms and are worthy of further exploration.

In summary, abnormal iron transport disrupts mitochondrial iron levels and ROS homeostasis, ultimately promoting ferroptosis.

#### 3.3.2 Iron utilization

HO-1 is an ER-anchored enzyme that metabolizes heme into oxidant-promoting ferrous iron, carbon monoxide and the antioxidant biliverdin^135^. HO-1 plays an essential role in clear cell renal cell carcinoma cell ferroptosis, and the mechanism involves the release of free iron mediated by HO-1-induced heme degradation, contributing to the generation of oxidized lipids in the mitochondrial membrane^136^, and it can be increased by the nuclear translocation of Nrf2^114^. This process is independent of the iron-regulating action of the canonical hepcidin/ferroportin axis. Similar results have been reported in studies of acute myeloid leukemia cells, human lung cancer cells, CRC cells and human breast cancer cells^77^, ^137^, ^138^, ^139^, ^140^. These data showed that high expression of HO-1 positively regulated ferroptosis through the modulation of iron and ROS levels, making it an important target for mediating the adverse effects of ferroptosis induction^141^. However, a previous study revealed that reduced HO-1 levels mediated synergistic lysosomotropic agent- and tyrosine kinase inhibitor-induced ferroptosis in glioblastoma cells^142^. Moreover, a decrease in HO-1 levels was not a consequence of decreased Nrf2 expression but due to proteasome degradation^142^. Similarly, HO-1 protected HCC and melanoma cells from ferroptosis^143^, ^144^, ^145^. This protective effect may be attributable to the HO-1 byproducts biliverdin and bilirubin; this effect was similar to that reported by another study in which GSH and bilirubin showed complementary antioxidant activities, and GSH protected water-soluble proteins and bilirubin protected lipids from oxidation^146^. In summary, these inconsistent results regarding the level of HO-1 expression observed during ferroptosis may be interpreted as context and cell type dependent.

Mitochondrial ISCs are modular cofactors that perform essential cellular functions. These clusters are synthesized via a multistep process involving the multisubunit mitochondrial machinery. The first step of ISC biosynthesis is the synthesis of [2Fe-2S] clusters on highly conserved scaffold proteins, with the disruption of this process increasing the iron load and promoting ferroptosis. For example, cysteine desulfurase 1 (NFS1) is an essential enzyme in eukaryotes that extracts sulfur from cysteine and makes it available for ISC biosynthesis, the suppression of which activates a robust iron starvation response mediated by iron-responsive element-binding protein 2 (IREB2, also known as IRP2); in conjunction with GSH synthesis inhibition, IREB2 activation drives ferroptosis *in vitro* and slows lung tumor growth^147^, ^148^. Moreover, frataxin and iron-sulfur cluster assembly enzyme (ISCU) are important proteins participating in this ferroptosis-inducing process, and their inhibition both significantly impedes ISC assembly and increases the ferroptosis rate by accelerating free iron accumulation in cancer cells^149^, ^150^, ^151^. Second, [2Fe-2S] is released from scaffold proteins and binds to transporters. Interference with this process affects ferroptosis sensitivity. For instance, glutaredoxin 5 (GLRX5) is an essential protein engaged in mitochondrial ISC transfer to IRP, m-aconitase, and ferrochelatase. The inhibition of GLRX5 expression predisposes cisplatin-resistant head and neck cancer cells to ferroptosis^152^. Third, the abnormal function of the ISC export machinery may cause ferroptosis. The IMM protein ABCB7 is a core component of the ISC export machinery and is directly involved in the export of ISCs synthesized in mitochondria. A recent study has reported that nonapoptotic cell death induced by ABCB7 depletion is partially mediated by ROS production triggered in response to changes in iron metabolism; however, additional characterization of this process is required to determine whether this death modality is, in fact, ferroptosis^153^. These data suggest that proteins involved in ISC assembly and export may play roles in ferroptosis by regulating iron metabolism.

#### 3.3.3 Iron storage

FtMt is an iron storage protein located in mitochondria that plays a significant role in modulating cellular iron metabolism. FtMt exerts a protective effect on ferroptosis triggered by erastin in SH-SY5Y neuroblastoma cells^154^, ^155^. Nuclear receptor coactivator 4 (NCOA4) is a selective cargo receptor that binds to ferritin and mediates its delivery to autophagosomes, which subsequently fuse with lysosomes, leading to ferritin degradation and concomitant iron release^156^. HERC2-mediated ubiquitylation of NCOA4 depends on iron, which ensures that the NCOA4 liberated from HERC2 under iron-deficient conditions contributes to ferritinophagy to replenish the iron pool^156^. Inhibition of NCOA4 suppresses ferritin degradation and ferroptosis in cancer cells^18^. In addition, the mRNA level of NCOA4 in macrophages is reduced under hypoxic conditions, thereby preventing ferroptosis, while NCOA4 and FtMt levels in fibrosarcoma cells do not respond to a decrease in the oxygen level^155^. In conclusion, blocking the binding of NCOA4 to FtMt, inactivating NCOA4, and reducing the stability of NCOA4 may protect cancer cells from ferroptosis by preventing ferritinophagy and maintaining the stored iron level.

### 3.4 Mitochondrial lipid metabolism in ferroptotic cells

**Figure 5:**
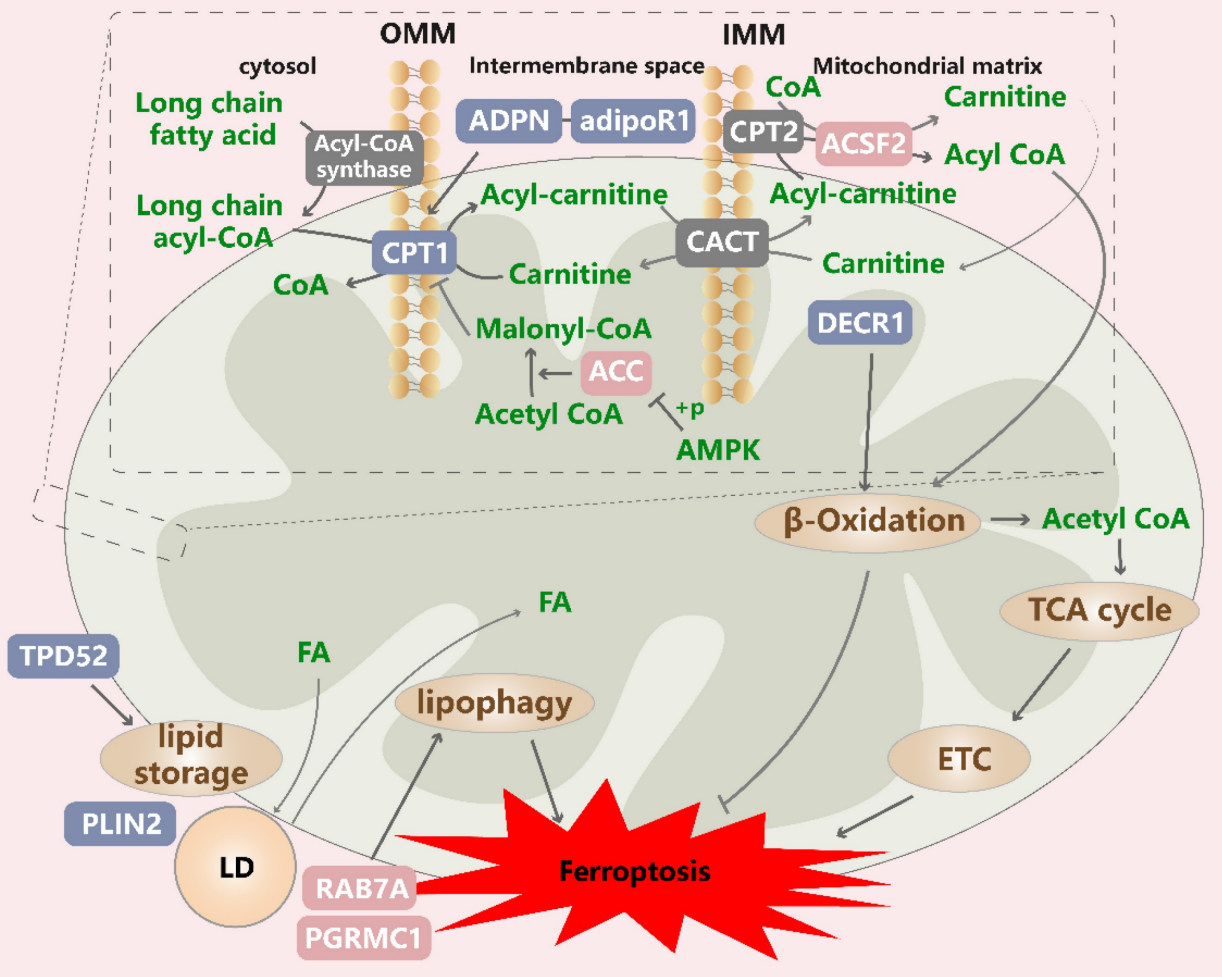
** Mitochondrial lipid metabolism in ferroptotic cells.** ACSF2 is encoded by a high-confidence gene and induces erastin-induced ferroptosis. CPT1/2 mediate the entry of long-chain acyl-CoA into mitochondria. CPT1 activity is inhibited by ACC-mediated malonyl-CoA production and restored by ADPN, affecting the ferroptosis sensitivity of cells. Increased ACC phosphorylation limits its activity. The DECR1-mediated promotion of polyunsaturated fatty acid (PUFA) β-oxidation confers ferroptosis resistance to cancer cells, but acetyl-CoA produced via fatty acid oxidation (FAO) enters the TCA cycle and affects ferroptosis sensitivity. The LD-specific marker PLIN2 and tumor protein D52, which are critical for lipid storage, exert protective effects on ferroptosis. In contrast, the expression of proteins involved in the fusion of LDs and mitochondria and subsequent lipophagy (RAB7A, PGRMC1) induces stored PUFA release and lipid peroxidation, leading to ferroptosis. ACSF2: acyl-CoA synthetase family member 2; CPT1/2: diacylglycerol cholinephosphotransferase 1/2; ADPN: adiponectin; DECR1: 2,4-dienoyl-CoA reductase 1; CACT/SLC25A20: solute vector family 25 member 20; TPD52: tumor protein D52; PLIN2: Perilipin2; PGRMC1: progesterone receptor membrane component 1.

The accumulation of lipid peroxides is a key step in ferroptosis (Figure 5). Initially, ACSF2 and CS were identified as high-confidence genes required for erastin-induced ferroptosis, and their silencing conferred broad protective effects on ferroptosis in a variety of cell lines^1^. Since these discoveries were made, research on mitochondrial fatty acid metabolism has also been reported. For example, we found that the role of mitochondrial fatty acid metabolism in ferroptosis is reflected mainly by the yield of specific lipid precursors needed for lipid peroxidation and substrates needed for energy metabolism (as described above).

First, a recently identified a specific lipid class, PC-PUFA_2_, has been shown to specifically interact with the mitochondrial ETC, enabling the initiation of ROS production in mitochondria and lipid peroxidation in the ER, thereby driving ferroptosis^30^. These fatty acids are catalyzed to produce acyl-CoA outside mitochondria. Long-chain acyl-CoA cannot directly permeate the IMM and thus is unavailable for oxidative decomposition; therefore, it is transported into mitochondria by L-carnitine. Carnitine palmitoyltransferase 1 (CPT1) is critical for this step, and its suppression enhances ferroptosis^111^, ^157^. When fatty acid oxidation (FAO) cannot proceed normally, other oxidative stress-related factors play relatively dominant roles, leading to fatty acid peroxidation. As an insulin-sensitizing hormone, adiponectin is an important factor in the regulatory networks involved in lipid metabolism and blood glucose homeostasis. Notably, adiponectin needs to bind to its cognate receptors to exert its biological effects. Adiponectin was shown to rescue ferroptosis induced by a fatty acid oxidation/peroxide imbalance by restoring CPT1 activity in individuals with gestational diabetes; however, whether this finding can be generalized to tumor cells remains to be investigated^158^. AdipoR1 is a verified adiponectin receptor, and its knockdown remarkedly inhibits the activation of the Nrf2/SLC7A11 pathway and increases the ferroptosis rate of HCC cells^159^. Malonyl-CoA, a substrate in fatty acid synthesis, is an inhibitor of CPT1 and is synthesized from ACC1/2^160^. Notably, increased ACC phosphorylation inhibits ACC activity, thereby protecting cells from ferroptosis, while its suppression inhibits ferroptosis driven by various inducers^95^, ^161^.

Next, acyl-CoA entering the mitochondrial matrix undergoes β oxidation, resulting in the formation of acetyl-CoA and acyl-CoA. 2,4-Dienoyl-CoA reductase 1 (DECR1) is the rate-limiting enzyme in a PUFA β oxidation auxiliary pathway, and its knockdown induces the significant accumulation of phospholipid hydroperoxides, increases the levels of mitochondrial oxidative stress, and induces ferroptosis in prostate cancer cells^162^. Another study revealed that DECR1 knockdown induces ER stress and sensitizes castration-resistant prostate cancer (CRPC) cells to ferroptosis, highlighting the importance of DECR1 in tumor progression and the development of therapeutic resistance^163^. In contrast, DECR1 knockdown does not affect saturated fatty acid and palmitate metabolism in mitochondria^162^. These results suggest that the induction of FAO can confer ferroptosis resistance to cancer cells, but the acetyl-CoA produced via FAO can enter the TCA cycle, where it interacts with other pathways to influence ferroptosis sensitivity.

Lipid droplets (LDs) protect cells from PUFA oxidation because free fatty acids are transferred to their hydrophobic core, thereby defending against oxidative stress^164^. This action may also explain the mechanism through which LDs inhibit ferroptosis. One study indicated that the overexpression of the LD-specific marker Perilipin2 (PLIN2) in gastric carcinoma cells increased number of LDs, reduced the intracellular ROS content and inhibited ferroptosis, which was interpreted as the relative hypoxia of cells due to a high lipid content, thereby inhibiting the occurrence of oxidative stress^165^. In addition, the inhibition of tumor protein D52, which is critical for lipid storage, decreased the number of LDs and increased the ferroptosis rate^166^. In contrast, lipophagy, which degrades intracellular LDs through autophagy, drives cytosolic fatty acids into mitochondria via the fusion of LDs and mitochondria, resulting in simultaneous FAO and LD formation in cells^167^, ^168^. Lipophagy has been reported to release stored PUFAs and induce lipid peroxidation and ferroptosis *in vivo*, which was inhibited by the knockdown of the LD cargo receptor RAB7A^166^, and induced by ionizing radiation^169^. Progesterone receptor membrane component 1 (PGRMC1) can alter lipid metabolism, promote autophagy and mediate chemotherapy resistance. One study showed that PGRMC1 affected lipophagy and FAO rates by anchoring LDs to mitochondria, and that its expression in paclitaxel-tolerant persister cancer cells increased the sensitivity to ferroptosis inducers^170^. These results suggest that the dynamic balance of LD formation and degradation exerts effects on ferroptosis. Notably, considering lipid metabolic reprogramming characterized by increased levels of free fatty acids, numbers of LDs and rate of FAO in resistant cancer cells, the modulation of LD formation and degradation may be a potential strategy to eradicate persister cells that survive conventional or targeted chemotherapy by increasing their susceptibility to ferroptosis^170^.

## 4. Clinical translation of antitumor effects

Traditional therapies have advantages and limitations, such as lower antitumor activity at low doses, toxic side effects due to off-target effects when applied at high doses, and the chemoresistance of tumor cells caused by adaptive tolerance. Ferroptosis, an adaptive mechanism to eliminate malignant cells, offers new possibilities for tumor suppression. Here, we outline several tumor therapeutic strategies based on mitochondria-associated ferroptosis-related mechanisms, which can be categorized as follows: first, targeting one or more ferroptosis targets within the mitochondria; second, a synergistic enhancement of efficacy by combining agents; and third, inducing ferroptosis can improve the efficacy of immunotherapy and enhance antitumor immune responses.

### 4.1 Targeting one or more ferroptosis biomarkers within the mitochondria

As mentioned above, agents target certain mitochondrial processes, such as GPX4, DHODH, iron metabolism pathway, etc., to increase intracellular oxidative stress or attenuate the ferroptosis defense system, thereby inducing the ferroptosis of tumor cells and blocking tumor cell growth. For example, commonly used ferroptosis inducers such as ML-162 and RSL3 reduce GPX4 activity to induce the ferroptosis of cancer cells and have been applied in the treatment of advanced treatment-resistant prostate cancer, ccRCC, HCC, TNBC and other cancers^171^, ^172^, ^173^, ^174^. Natural product derivatives, synthetic drugs and nanoparticle-encapsulated molecules have also been extensively studied and shown to exert antitumor effects by affecting GPX4 translation, proteolysis and protein modification^173^, ^174^, ^175^, ^176^, ^177^, ^178^. Notably, Gan H, *et al.* developed a theranostic ferroptosis inducer (IR780-SPhF) that enables the simultaneous imaging and therapy of TNBC by directly targeting mitochondria to monitor and deplete mitochondrial GSH levels. IR780-SPhF exhibited stronger anticancer potency than cyclophosphamide, indicating that mitochondria-targeted ferroptosis inducers may represent promising candidates for precision cancer therapy^179^, ^180^.

Moreover, a few Chinese herbs, engineered compounds and autophagy modulators have been characterized as ferroptosis inducers that are able to enhance mitochondrial dysfunction, thereby exerting anticancer effects on NSCLC, HCC, breast cancer stem cells^58^, ^180^, ^181^, ^182^, ^183^, ^184^. For example, andrographolide (ADE), a major effective ingredient of *Andrographis paniculata*, has been shown to increase mtROS release, MMP depolarization, mitochondrial ATP reduction and ferroptosis, repressing NSCLC cell growth, which could be rescued by a mitochondria-targeted antioxidant^181^. These results emphasize the involvement of mitochondrial oxidative stress and disrupted energy metabolism in the induction of ferroptosis-mediated anticancer effects by these agents.

Additionally, novel mitochondria-targeted nanosystems have played an eye-opening role in precision cancer therapy. These nanosystems efficiently deliver significant amounts of CO, iron, ferroptosis inducers, and photosensitizers to mitochondria to induce mtROS production and finally drive ferroptosis, and can be utilized in combination with nonclassical physiotherapy^185^, ^186^, ^187^, ^188^, ^189^.

### 4.2 Synergistic enhancement of efficacy by combining agents

Combination therapy shows promise in overcoming compensatory mechanisms and unwanted off-target effects. For example, brequinar has been reported to disrupt the ferroptosis defense system of GPX4_low_ tumors, inhibiting their growth by inhibiting mitochondrial DHODH. For GPX4_high_ tumors, DHODH inhibition increased cellular GSH levels, thus making GPX4 more effective in suppressing ferroptosis^98^. Brequinar has been tested in large clinical trials for treating advanced melanoma, gastrointestinal cancer, and squamous cell carcinoma of the head or neck^190^, ^191^, ^192^. The combination of DHODH inhibitors with sulfasalazine, oxaliplatin and cisplatin has been shown to sensitize cells to ferroptosis and reverse chemoresistance^98^, ^104^, ^193^. Moreover, the therapeutic agent NL-1, an inhibitor of the mitochondrial iron transporter CISD1, can accelerate sorafenib-induced lipid peroxidation and ferroptosis by promoting mitochondrial iron accumulation, which increases the efficacy of sorafenib in HCC^194^. Notably, several iron-containing nanoparticle formulations have been developed to load ferroptosis inducers into tumors, accelerating the destruction of antioxidant systems and the accumulation of lipid peroxides, resulting in the synergistic inhibition of tumor growth^195^, ^196^.

Some unconventional pathway regulators have also shown promising efficacy in combination with ferroptosis inducers, and they usually act synergistically upstream and downstream. Xue, Xiangfei *et al.* reported that FDA-approved c-Jun N-terminal kinase (JNK) agonists reinforced RB1CC1 nuclear translocation and sensitized cells to ferroptosis. Coadministering imidazole ketone erastin with four different JNK activators in mouse models resulted in an increase in anti-tumorigenic efficacy^83^. The combination with autophagy activators has also been described to play a role in sensitizing cells to the anticancer potency of ferroptosis inducers, given that the autophagy-ferroptosis-ROS-autophagy loop has been described as an amplifier of ferroptosis under certain conditions^82^, ^197^. For example, rapamycin alleviated the blockade of autophagy flux induced by COX7A1, further increasing the lethality of ferroptosis inducers in NSCLC cells^82^.

In contrast to normal cells, most cancer cells exhibit increased aerobic glycolysis and impaired oxidative phosphorylation, also known as the Warburg effect^198^. In combination with 2DG, which mimics glucose starvation, the antiglioma effect of chloramphenicol was enhanced, which disrupted mitochondrial ribosomes and triggered mitochondrial oxidative stress and ferroptosis^199^. This result may be because the glucose starvation state makes mitochondria dominant. This mechanism by which metabolic reprogramming enhances the antitumor effects of combination therapy could provide new insights into the development of new combination regimens.

In addition, the mitochondria-targeted nanodelivery system mentioned above combined with photodynamic and sonodynamic physiotherapy is expected to achieve simultaneous monitoring and treatment.

### 4.2 Inducing ferroptosis can improve the efficacy of immunotherapy and enhance antitumor immune responses

In recent years, immune checkpoint blockade (ICB) has been a breakthrough strategy for the treatment of cancers^200^. On one hand, ferroptosis shows potential for increasing the responsivity of ICB by inducing immunogenic cell death (ICD)^201^. On the other hand, triggering ferroptosis in the tumor microenvironment to deplete immunosuppressive cells can also reverse immunotherapy resistance. Specifically, during ferroptosis, intracellular damage-associated molecular patterns (DAMPs), such as calreticulin (CRT), adenosine triphosphate (ATP), high mobility group box 1 (HMGB1), and mtDNA, accumulate or are relocalized^201^. The increased levels of DAMPs in the tumor microenvironment could further enhance ICB. Therefore, amplifying the effect of ferroptosis to drive DAMP production could be a viable strategy to induce ICD and enhance the antitumor immunity of ICB^202^. For example, manganese has been reported to induce ferroptosis in cancer cells through type-I IFN-dependent inhibition of mitochondrial DHODH. A recent study showed uncontrolled tumor growth and metastasis in Mn-deficient mice, while the cellular cGAS-STING pathway was activated after Mn supplementation, which increased antigen delivery and increased the activation of CD8^+^ T cells and NK cells to exert antitumor effects^203^. Similarly, mitochondrial-targeting liposomes and nanoparticles have also been elaborately developed for drug delivery, activating the cGAS-STING pathway and triggering ICD. Their antitumor activity has been observed in bladder cancer and lung cancer^202^, ^204^. These data suggest that ferroptosis promotes the specific activation of STING pathway represents a potent strategy to increase the efficacy of immunotherapy in tumors. Additionally, previous studies have fabricated cinnamaldehyde dimers (CDC) into lipid-like materials to form dimersomes, which deplete GSH and deliver therapeutic agents to enhance ferroptosis and the efficacy of immunotherapy in breast cancer^205^. Yang F, *et al.* suggested that the combination of GPX4 inhibitors and anti-PD1 possessed greater therapeutic efficacy than the monotherapy for tumors resembling LAR in biological characteristics, which was attributed to LAR tumors eminently utilizing the GSH system for detoxification^29^.

In addition to activating tumor cell immunogenicity, the inhibition of tumor cell immune escape also helps to enhance the immune response. Targeting PD-1/PD-L1 is a common strategy used in ICB. Researchers found that the mitochondrial translocator protein (TSPO) inhibits ferroptosis by enhancing the Nrf2-dependent antioxidant defense system and promotes HCC immune evasion by upregulating PD-L1 expression through Nrf2-mediated transcription. Notably, the TSPO inhibitor PK11195 administrated in combination with anti-PD-1 antibody exerted a synergistic antitumor effect on a mouse model^206^. Moreover, the active molecule B2 derived from a novel mitochondrion-targeting DHODH inhibitor can downregulate PD-L1 expression and alleviate immunosuppression by decreasing the expression of the mitochondria-related protein MTHFD2, thereby promoting ferroptosis in melanoma cells^207^.

In addition to tumor cell-intrinsic approaches, triggering the ferroptosis of immune suppressor cells in the tumor microenvironment is a viable approach to increase immunotherapy efficacy. A study of melanoma showed that tumor growth was suppressed in mice with a Treg-specific deletion of GPX4 but antitumor immune responses were enhanced without inducing autoimmunity^208^. In a study of lung cancer, researchers developed zero-valent-iron nanoparticles (ZVI-NPs), which can simultaneously target the tumor microenvironment and cancer cells. Specifically, they accumulate preferentially in tumor and lung tissues, causing mitochondrial dysfunction and ferroptosis in lung cancer cells, and target the iron homeostasis of immune cells. ZVI-NPs significantly regulated macrophage polarization from the immunosuppressive M2 phenotype to the antitumor M1 phenotype and decreased the proportion of Tregs, augmenting antitumor immunity^189^. To date, several nanoparticles have been shown to inhibit tumor growth by reprogramming the TME and promoting tumor ferroptosis^209^, ^210^. Therefore, the induction of ferroptosis in immunosuppressive cells might be an innovative approach to potentiate the efficacy of immunotherapy.

The insufficient immunogenicity of tumor cells, sensitization of immune cells to ferroptosis by oxidative stress, and immunosuppressive TME are the causes of the poor response to tumor immunotherapy. Therefore, based on the biological characteristics of the immune TME, researchers can determine whether the application of combinations of immunotherapy and ferroptosis-targeting therapies is appropriate to improve efficacy.

## 5. Concluding remarks

Research into the mechanism underlying the promotion lipid peroxidation and ferroptosis and the sophisticated molecular mechanism regulating the antioxidant defense system has led to a greatly improved understanding. Interference with ferroptosis via key regulators exerts potential therapeutic effects on cancer cells. The discovery of a complex metabolic regulatory network and the pluripotency of ferroptosis inhibitors with different targets has established a blueprint for us to use in developing therapies.

Glucose, lipids, amino acid and iron metabolism, which provide energy for tumor cells to support growth and maintain iron homeostasis, are mitochondrial processes. When the energy status is abnormal in tumor cells or these cells are exposed to exogenous stimuli, a variety of metabolic activities in mitochondria may cause oxidative stress and promote ferroptosis^13^, ^95^. However, mitochondria are equipped with potent antiferroptotic defense factors, such as GPX4^mito^ and DHODH-derived CoQ^25^. An impaired mitochondrial antiferroptotic defensive system cannot eliminate lipid peroxides, which can accumulate to lethal levels in mitochondria, and this imbalance between oxidative stress and the antioxidant system can lead to ferroptosis^102^. In summary, because of metabolic abnormalities and damage to mitochondria during ferroptosis in different models, an increasing number of studies on mitochondrial quality control pathways have proposed that mitochondria are intricately involved in the regulation of ferroptosis.

Mitochondria link ferroptosis with other classical death modalities, such as autophagy and apoptosis, suggesting a new perspective for exploring the complex mechanisms underlying various death modalities. For example, the aforementioned VDACs can be antagonized by erastin, resulting in channel opening, an elevated MMP, increased mtROS generation and ferroptosis^211^. Previous studies have shown that VDACs are key proteins involved in the mPTP for mitochondria-mediated apoptosis, facilitating the influx of proapoptotic proteins into cytosol via mitochondrial channels^211^. These effluents, such as mtDNA, are sensed by the cGAS/STING pathway and initiate inflammatory signaling and immune responses^212^. *In situ* Raman spectroscopy revealed that erastin may induce apoptosis and accelerate the release of Cyt c from mitochondria. However, Cyt c can trigger ferroptosis by initiating Fenton-like reactions and lipid peroxidation^213^. In addition, recent studies have found that iron in melanoma cells acts as a sensitizing agent for ROS, and the induced oxidation of the OMM protein TOM20 recruits Bax to mitochondria, thereby triggering subsequent Caspase3-GSDME-mediated pyroptosis^214^. Indeed, iron can trigger different types of cell death, depending on the activation of different signaling pathways. Moreover, Hsp90 has been identified as a coregulatory node of necroptosis and ferroptosis. The necroptosis inhibitor CDDO inhibits necroptosis and ferroptosis by targeting HSP90^215^. Another study showed that RIPK3, a key molecule involved in necroptosis, has a dual role when responding to drug-induced organ damage. It not only phosphorylates MLKL to induce necroptosis but also phosphorylates FSP1 to inhibit its enzymatic activity, thereby promoting ferroptosis^216^. Moreover, RIPK3 can promote mitochondrial energy metabolism and upregulate the expression of mitochondrial NADPH oxidase 4 to induce mitochondrial damage^217^. The simultaneous occurrence of necroptosis and ferroptosis may be related to acute tissue injury. Studies have shown that autophagy is involved in various pathways of ferroptosis (ferritinophagy, lipophagy, chaperone-mediated autophagy, etc.). Autophagy activates ferroptosis by selectively degrading antiferroptotic factors or increasing substrates to promote the accumulation of lipid peroxides^218^, ^219^. On the other hand, oxidative stress and lipid peroxidation products are powerful inducers of autophagy, while excessive autophagy further amplifies the ferroptosis signal^218^, ^219^. As a key protein involved in various signal transduction pathways, AMPK activates autophagy by negatively regulating mTOR. Several inhibitors and drugs can induce ferroptosis and autophagy by inhibiting mTOR or activating the AMPK pathway, as mentioned above; rapamycin further enhances the anticancer efficacy of ferroptosis inducers^82^. Taken together, since proteins and signal transduction pathways are shared between different cell death pathways, they provide subtle and plastic control of the cell fate in response to specific stresses, thus providing opportunities for therapeutic interventions to direct the cell death response.

Researchers have attempted to translate the current research on the mechanism by which mitochondria regulate cancer-related ferroptosis into clinical applications to address the issues of drug resistance, off-target effects, and the side effects of traditional chemotherapy or immunotherapy. Mitochondrial function is closely related to processes such as stress, metabolic reprogramming, and autophagy. Therefore, the corresponding regulators could work together with ferroptosis inducers in cancer therapy. The combination of ferroptosis modulators and immunotherapy is a highly discussed strategy for cancer treatment. However, its clinical translation is worrisome due to its double-edged sword effect. Ferroptosis may target both tumor cells and antitumor immune cells. Therefore, tailoring ferroptosis-related therapies to the heterogeneity of ferroptosis in different cancer cells, elucidating TME properties suitable for this therapy, and developing more specific targeted therapies may provide important insights into the design of effective combinatorial strategies against cancer.

The limitations are mentioned above. First, the intricate and dynamic network of mitochondrial regulation of ferroptosis involves multiple pathways, and thus identifying the dominant pathway according to the properties of the cell can help in the design of targeted therapeutic strategies. Second, although a variety of therapeutic approaches are under development, most of them have only been tested in animal experiments rather than large-scale clinical trials, and thus much more work is needed before clinical translation.

## Abbreviations

ROS: reactive oxygen
ALOX: lipoxygenase
POR: cytochrome P450 oxidoreductase
TFR1: transferrin receptor 1
FTL: ferritin light chain
FTH1: ferritin heavy chain 1
FPN1: ferroportin
PROM2: Prominin2
GSH: glutathione
Glu: glutamate
GPX4: glutathione peroxidase 4
FSP1: ferroptosis suppressor protein 1
AIFM2: apoptosis-inducing factor mitochondria-associated 2
CoQ: coenzyme Q10
AR: androgen receptor
PUFAs: polyunsaturated fatty acids
ACSL4: acyl-CoA synthetase long-chain family member 4
LPCAT3: lysophosphatidylcholine acyltransferase 3
ETC: electron transport chain
NADPH: nicotinamide adenine dinucleotide phosphate
MPO: myeloperoxidase
PDAC: pancreatic ductal adenocarcinoma
TRAP1: tumor necrosis factor receptor-associated protein 1
HSP90: heat shock protein 90
DRP1: dynamin-related protein 1
Fis1: fission 1
PINK1: PTEN-induced kinase 1
NSCLC: non-small-cell lung cancer
MTS: mitochondrial targeting sequence
OPA1: optic atrophy 1
MFN: mitofusin
ATF4: activating transcription factor 4
PHB2: prohibitin 2
IMM: inner mitochondria membrane
ISCs: iron-sulfur clusters
HO-1: heme oxygenase-1
CRC: colorectal cancer
TFAM: transcription factor A
COX7A1: cytochrome c oxidase subunit 7a
PGC-1α: mitochondrial biogenesis activator
CHCHD3: coiled-coil-helix-coiled-coil-helix domain containing 3
MTCH1: mitochondrial carrier 1
ACC: acetyl CoA carboxylase
PC: pyruvate carboxylase
IDH2: isocitrate dehydrogenase
α-KG: α-ketoglutarate
Gln: glutamine
MPC1: mitochondrial pyruvate carrier 1
AMPK: AMP-activated protein kinase
MEFs: embryonic fibroblasts
BECN1: beclin 1
VDACs: voltage-dependent anion-selective channels
H_2_O_2_: hydrogen peroxide
MDA: malondialdehyde
G3P: glycerol-3-phosphate
LOX: lysyl-oxidase
AK2: adenylate kinase 2
ER: endoplasmic reticulum
PLs: PUFA-phospholipids
DIC: dicarboxylate carrier
OGC: oxoglutarate carrier
NAC: N-acetylcysteine
HCC: hepatocellular cancer
DHLA: reduced form dihydrolipoic acid
TXN: thioredoxin
ARE: antioxidant-response element
OMM: outer mitochondrial membrane
FtMt: mitochondrial ferritin
Mfrn: mitoferrin
DMT1: divalent metal transporter 1
NFS1: cysteine desulfurase 1
IRP2: iron-responsive element-binding protein 2
ISCU: iron-sulfur cluster assembly enzyme
GLRX5: glutaredoxin 5
NCOA4: nuclear receptor coactivator 4
CPT1: carnitine palmitoyltransferase 1
FAO: fatty acid oxidation
DECR1: 2,4-Dienoyl-CoA reductase 1
CRPC: castration-resistant prostate cancer
LD: lipid droplet
PLIN2: Perilipin2
PGRMC1: progesterone receptor membrane component 1
ICB: immune checkpoint blockade
ICD: immunogenic cell death
DAMPs: intracellular damage-associated molecular patterns
ATP: adenosine triphosphate
HMGB1: high mobility group box 1
